# Complement and transplant‐associated thrombotic microangiopathy: Current and future approaches

**DOI:** 10.1002/hem3.70370

**Published:** 2026-04-23

**Authors:** Massimo Cugno, Francesco Onida, Bernhard Lämmle

**Affiliations:** ^1^ Department of Pathophysiology and Transplantation Università degli Studi di Milano Milan Italy; ^2^ Department of Internal Medicine, SC Medicina‐Emostasi e Trombosi Fondazione IRCCS Ca' Granda Ospedale Maggiore Policlinico Milan Italy; ^3^ Haematology and BMT Unit, ASST Fatebenefratelli‐Sacco, Department of Oncology and Haemato‐Oncology Università degli Studi di Milano Milan Italy; ^4^ Center for Thrombosis and Hemostasis, University Medical Center Johannes Gutenberg University Mainz Germany; ^5^ University Clinic of Hematology and Central Hematology Laboratory, Inselspital, Bern University Hospital University of Bern Bern Switzerland

Transplant‐associated thrombotic microangiopathy (TA‐TMA) is a severe complication of allogeneic hematopoietic stem cell transplantation (allo‐HSCT) characterized by platelet consumption, microangiopathic hemolytic anemia, and multi‐organ dysfunction.^1^ Renal involvement is common and often results in hypertension. TA‐TMA affects both children and adults^2^, ^3^, ^4^ and typically arises in the early post‐transplant period (usually within 100 days), a phase marked by incomplete marrow recovery and frequent complications such as infections, immune‐mediated injury, and drug toxicity. These overlapping features complicate diagnosis. Histological confirmation, although the diagnostic gold standard, is rarely pursued due to bleeding risk, and several clinical criteria have therefore been proposed.^5^ Due to differences in diagnostic criteria,^6^ the epidemiology of TA‐TMA remains poorly defined, and its incidence varies from 3% to 39%. The case‐fatality rate is also imprecisely defined and has been reported to be as high as 80%.^3^ A recent multicenter prospective study found severe TA‐TMA in 21.8% of 239 patients within 100 days from the allo‐HSCT; these patients showed a higher nonrelapse mortality (42%) than those with nonsevere (8.4%) or no TA‐TMA (5.8%).^7^

Recent advances have reframed TA‐TMA from a poorly defined complication to a biologically coherent syndrome with evolving diagnostic frameworks and expanding therapeutic options. Progress stems from two main developments: (1) a converging, evidence‐based “multi‐hit” pathophysiologic concept placing endothelial injury and complement activation at the center of disease biology, and (2) consensus efforts to harmonize diagnostic and response criteria, both of which are prerequisites for meaningful clinical trials and consistent care.

## COMPLEMENT SYSTEM AND TA‐TMA PATHOPHYSIOLOGY

TA‐TMA is best conceptualized as a multi‐hit process involving genetic or acquired predisposition to enhanced complement activation (hypofunctional mutations of complement regulatory proteins, hyperfunctional variants of complement factors, and endothelial vulnerability), exposure to endothelial stressors (conditioning regimens, calcineurin inhibitors, infections, and high cytokine load), and ongoing inflammatory/immune drivers (acute graft‐versus‐host disease (GVHD), viral reactivation). These factors converge on the microvascular endothelium, yielding activation of the complement system and activation of hemostasis with resultant thrombosis of the microcirculation.^8^

### Complement system

**Figure 1:**
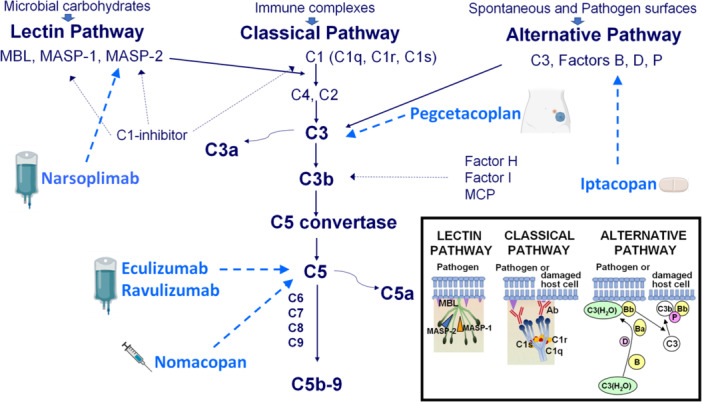
**Simplified diagram of the complement system and the target sites of drugs used in patients with transplant‐associated thrombotic microangiopathy.** Complement activation occurs through three pathways: the classical pathway (triggered by antibody–antigen complex), the alternative pathway (spontaneously activated at a low level or triggered by specific surface antigens), and the lectin pathway (activated by binding mannose residues on the pathogen surface). The classical pathway is initiated by the effect of antibody–antigen complex on the three components of C1, that is, C1q and the two proteases C1r and C1s. After the sequential activation of C1r and C1s, activated C1s cleaves C4 and C2, leading to the formation of the C3 convertase C4b2b (also called C4b2a according to the old terminology). This enzymatic complex cleaves C3 into the opsonin C3b and the anaphylatoxin C3a. The lectin pathway is similar to the classical pathway, except that it is initiated by the pattern recognition receptors mannose‐binding lectin (MBL), ficolins, and collectins. These molecules recognize and bind predominantly to specific carbohydrate structures present on microbial surfaces. The binding of MBL to the pathogen surface induces the activation of its two associated serine proteases, that is, MBL‐associated serine protease‐1 (MASP‐1) and MASP‐2, which are sequentially activated. MASP‐2 cleaves C4 and C2 to form the same C3 convertase as in the classical pathway (C4b2b). The activation of the classical and lectin pathways is controlled by C1‐inhibitor that can block C1r, C1s, MASP‐1, and MASP‐2. The alternative pathway is continuously active at a low level through spontaneous hydrolysis of C3 that binds Factor B, which is then cleaved by Factor D to form the soluble initial C3 convertase C3(H_2_O)Bb. This enzyme complex can cleave additional C3 molecules, generating C3b that covalently attaches to activating surfaces (pathogen membranes, altered self, or damaged tissue). When bound to a surface, C3b is protected from inhibition, and this bound C3b recruits more Factor B and Factor D, generating the alternative pathway C3 convertase C3bBb. The stabilizing protein properdin also binds to the alternative C3 convertase, forming a more stable complex, C3bBbP, which can further cleave C3 into C3a and C3b and greatly amplifies the complement activation. The alternative pathway is regulated by soluble inhibitors such as Factor H and Factor I as well as by cell‐bound inhibitors such as membrane cofactor protein (MCP). Factor H binds to C3b and inhibits some of its functions. It also acts as a cofactor for Factor I, which proteolytically cleaves and permanently degrades C3b. The activation of the three pathways (classical, lectin, and alternative) converges on the common pathway, known as “terminal pathway” in which the formation of C3 convertases facilitates the generation of C5 convertases through additional binding of C3b molecules, forming C4b2b3b (from the classical or lectin pathways) or C3bBb3b (from the alternative pathway). These C5 convertases cleave C5 into the anaphylatoxin C5a and the C5b molecule, the latter initiating the formation of the membrane attack complex (MAC; C5b‐9) that leads to the lysis of target cells. Several drugs can inhibit the activation of the complement system. C5 is blocked by eculizumab (intravenous infusion over 25–45 min), ravulizumab (intravenous infusion ranging from 0.4 to 3.8 h), or nomacopan (subcutaneous administration). MASP‐2 is inhibited by narsoplimab (30‐min intravenous infusion). The activation of C3 can be blocked by pegcetacoplan (30‐min subcutaneous infusion if using two sites or 60‐min subcutaneous infusion if using one site). Finally, Factor B is inhibited by iptacopan (orally administered).

The complement system comprises proteins that activate sequentially in a regulated cascade.^9^ Activation, which occurs through the classical, lectin, or alternative pathways (Figure 1), contributes to innate immunity, generating chemotactic and vasoactive peptides that promote inflammation, opsonization, and producing bacterial cell lysis via the terminal C5b‐9 complex. Complement also aids the clearance of apoptotic and necrotic cells.

#### Classical pathway

The classical pathway is initiated when C1q, a hexameric pattern‐recognition molecule, binds to the Fc regions of antibodies (typically IgM or IgG1/IgG3) that have complexed with antigen, or directly to certain pathogens or apoptotic cell surfaces. Binding activates the associated serine proteases C1r and C1s, forming the C1 complex. Activated C1s cleaves C4 into C4a and C4b, the latter covalently attaching to the activating surface via its thioester bond. C2 then binds to surface‐bound C4b and is cleaved by C1s to generate C4b2b, the classical C3 convertase, which efficiently cleaves C3 into C3a (an anaphylatoxin) and C3b, a powerful opsonin.

#### Lectin pathway

The lectin pathway mirrors the classical cascade but uses non‐antibody pattern‐recognition molecules, principally mannose‐binding lectin (MBL), which recognizes carbohydrate or acetylated motifs on microbial surfaces. MBL circulates in complex with the MBL‐associated serine proteases (MASP‐1 and MASP‐2). Upon binding to target surfaces, MASP‐2 becomes activated and cleaves C4 and C2, forming the same C3 convertase as in the classical pathway (C4b2b). Thus, both classical and lectin routes converge at C3 activation through a similar enzymatic machinery.

#### Alternative pathway

The alternative pathway is continuously active at low level through spontaneous hydrolysis of the internal thioester of C3, producing C3(H_2_O), the so‐called tick‐over, allowing a prompt response upon microorganism challenge. The fluid‐phase C3(H_2_O) binds Factor B, which is then cleaved by Factor D to form the soluble initial C3 convertase C3(H_2_O)Bb. This enzyme can cleave additional C3 molecules, generating C3b that covalently attaches to nearby surfaces. On activating surfaces (pathogen membranes, altered self cells, or damaged tissue), bound C3b recruits Factor B and forms C3bBb, the final alternative pathway C3 convertase, which is stabilized by properdin (Factor P). When bound to a surface, C3b is protected from the action of inhibitors, and this bound C3b recruits more Factor B, Factor D, and Factor P, and greatly amplifies the complement activation. The alternative pathway functions as a self‐amplifying “amplification loop” for the entire complement system.

#### Terminal pathway

All three complement pathways converge at C3, producing C3b molecules that can attach to surfaces and combine with existing C3 convertases to form C5 convertases, that is, C4b2b3b (classical/lectin) and C3bBb3b (alternative). These enzymatic complexes cleave C5 into C5a, a potent proinflammatory and chemoattractant anaphylatoxin, and C5b, which initiates assembly of the terminal complement complex. Sequential binding of C6, C7, C8, and C9 to C5b forms the C5b‐9 membrane attack complex (MAC). The MAC creates transmembrane pores that disrupt osmotic balance, leading to lysis of susceptible cells such as Gram‐negative bacteria or complement‐sensitive host cells.

#### Complement control

Tight control by regulatory proteins prevents excessive activation and host cell damage, ensuring that complement activity remains localized to “danger surfaces.” Complement inhibitors may act in the fluid phase, like C1‐inhibitor, Factor H and Factor I, or as membrane‐bound factors, like CD46 (cluster of differentiation 46), also known as membrane cofactor protein (MCP).

### Complement involvement in TA‐TMA

**Figure 2:**
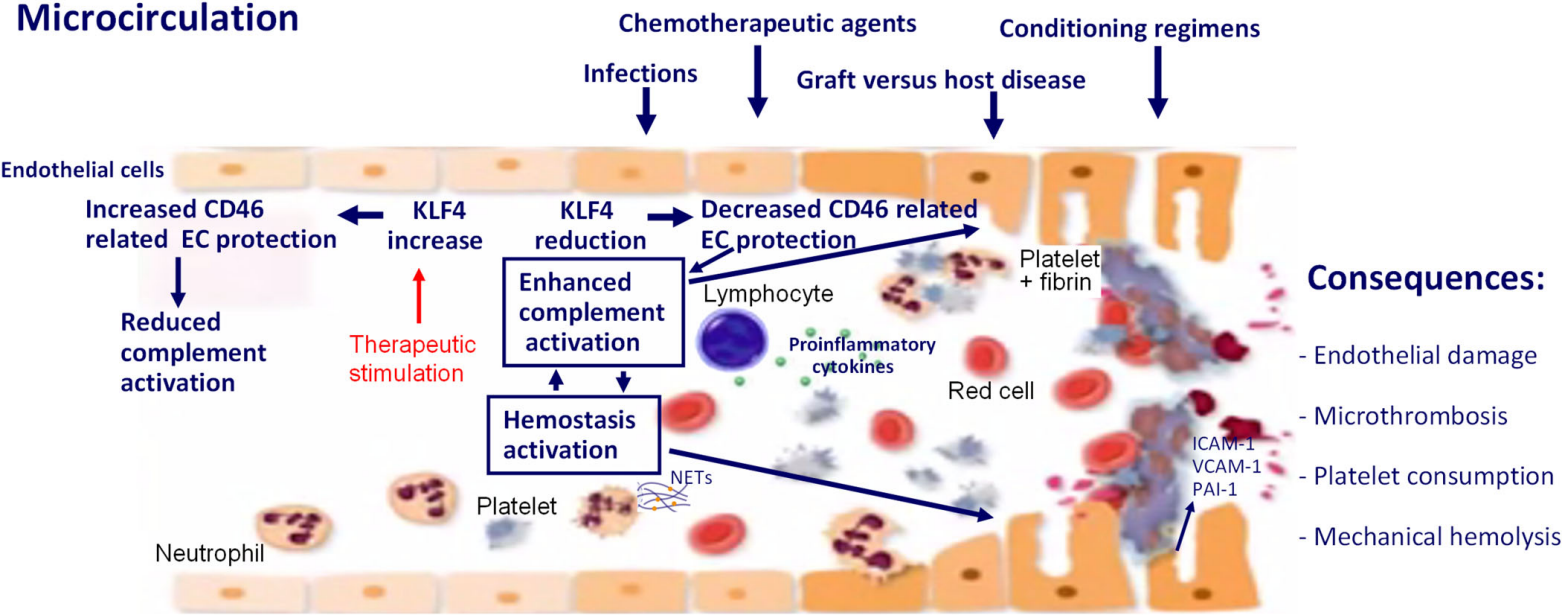
**Pathophysiology of transplant‐associated thrombotic microangiopathy.** Infections, chemotherapeutic agents, graft‐versus‐host disease, and conditioning regimens lead to endothelial injury and to an increase in proinflammatory cytokines, prothrombotic factors, and complement activation, which in turn further damage the endothelium. A predisposition to complement hyperactivation may be due to the presence of variants of complement system regulatory proteins. Neutrophil extracellular traps (NETs) link endothelial injury and complement activation. The reduced plasma levels of KLF4 leading to low expression of CD46 (complement inhibitor also known as membrane cofactor protein [MCP]) render the endothelium more vulnerable to complement activation.

The same properties making complement an efficient defense mechanism render it potentially deleterious when dysregulated or excessive. When regulators are overwhelmed, inactivated, or absent (cytokine storm, oxidative stress, endothelial injury, or presence of genetic variants), unrestrained complement activation can damage host tissue, and microvascular endothelium is particularly vulnerable.^8^, ^9^ When exposed to inflammatory or oxidative stressors as in allo‐HSCT, endothelial cells lose surface regulators and upregulate adhesion molecules and procoagulant factors (Figure 2). Conditioning toxicity, calcineurin/mTOR inhibitors, infections (e.g., CMV), and acute GVHD generate a pro‐thromboinflammatory milieu.^8^ This primes complement on the microvascular surface. In several patients, inherited variants in complement genes (loss‐of‐function variants of CFH, CFI, MCP, and gain‐of‐function variants of Factor B and C3) lower the threshold for disease, explaining variable susceptibility and severity. Another mechanism involves reduced levels of the transcription factor Krüppel‐like Factor 4 (KLF4) leading to low expression of CD46, which renders the endothelium more vulnerable to complement activation in TA‐TMA.^10^ The possibility of pharmacologically increasing KLF4 levels with the subsequent increase of endothelial CD46 expression can protect against complement hyperactivation and endothelial injuries in TA‐TMA^10^ and potentially in other endothelium‐related complications of allo‐HSCT.^11^

## DIAGNOSTIC AND RESPONSE CRITERIA OF TA‐TMA

To harmonize diagnostic approaches, the major international transplant societies recently endorsed a consensus definition for TA‐TMA,^12^ representing an important advancement in the field. This consensus, adapting the criteria developed by Jodele et al for the pediatric population,^2^ defines that TA‐TMA is diagnosed when at least four of the following seven features are present twice within 14 days: anemia, thrombocytopenia, hypertension, increased lactate dehydrogenase (LDH), schistocytes, soluble C5b‐9, and proteinuria.^12^ These criteria facilitate early diagnosis of TA‐TMA that is essential for timely therapy and provide a standardization that is useful for trials and registries. Soluble C5b‐9 testing is encouraged when available, but the consensus recognizes that access to testing may be limited but important to decide for a targeted therapy such as complement inhibition. An external validation confirmed that TA‐TMA remains a significant contributor to non‐relapse mortality following allo‐HSCT.^13^

The same expert panel extended its mandate to review response criteria.^14^ Three principles were emphasized. First, complete resolution may be delayed due to patient complexity, making partial response (PR) assessments essential for early treatment evaluation. Second, hematologic and biochemical abnormalities often recover faster than organ dysfunction; thus, these domains should be evaluated separately alongside overall response. Third, objective response definitions were proposed for each diagnostic feature and organ system. Hematologic and biochemical assessments include hemoglobin, platelet count, schistocytes, LDH, soluble C5b‐9, and transfusion dependence, while organ‐specific responses address kidney, lung, gastrointestinal, and central nervous system involvement. Overall response is determined by the least improved component. Although consensus was reached on definitions, standardized timing for response evaluation remains undetermined. These criteria aim to unify response assessment, enhance comparability across studies, and guide future therapeutic evaluation in TA‐TMA. In addition, routine and specific laboratory biomarkers may assist diagnosis, severity assessment, and treatment optimization.^15^ In particular, elevated soluble C5b‐9 has diagnostic and prognostic relevance. For testing sC5b‐9, pre‐analytical conditions are critical in order to prevent in vitro activation of both complement and coagulation, which are strictly linked and can activate each other. In our experience, blood samples should be collected by clean venipuncture from an antecubital vein or from a central venous catheter in tubes containing ethylenediaminetetraacetic acid (EDTA) 0.13 mol/L, which chelates calcium and efficiently blocks both complement and coagulation activation. Within 2 h, the tubes should be centrifuged at 2000 × *g* for 10 min at room temperature, and the plasma aliquots are stored in polystyrene tubes at −80°C until testing. Several ELISA methods are available for plasma measurement of sC5b‐9, and some of them have been approved for diagnostic use in Europe, labeled as CE‐IVD (In Vitro Diagnostic), whereas others have been approved only for research. Proteinuria (random urine protein/creatinine ≥ 1 mg/mg) is a hallmark of TA‐TMA‐associated kidney injury in children and young adults.^2^, ^15^ A rapid and acute increase in blood pressure with other concurrent signs or symptoms of microangiopathy supports a diagnosis of TA‐TMA. Several complement‐related biomarkers are available; however, they have moderate specificity individually, but longitudinal monitoring improves diagnostic accuracy and may guide therapy initiation, adjustment, and response evaluation.^15^

## CURRENT STANDARD OF CARE: SUPPORTIVE AND COMPLEMENT TARGETED THERAPIES

Various therapies have been proposed over the years, but few have proved consistently effective. Plasma exchange and defibrotide, a mixture of single‐stranded oligonucleotides with endothelial protective properties, have shown efficacy in selected cases. Currently, management combines supportive care with targeted therapy. Supportive care consists of a rigorous control of blood pressure, transfusion support, judicious adjustment of potentially offending drugs (e.g., shift from calcineurin inhibitors to other immunosuppressants or reassessing their dosing), aggressive infection treatment, and organ‐support as needed. These measures are foundational and accompany the targeted therapy, which, at present, is aimed to counteract complement activation. Complement blockade has emerged as the most important specific intervention for severe TA‐TMA. Jodele et al. studied 21 children with TA‐TMA treated with eculizumab and demonstrated a survival of 71% 6 months after diagnosis and 62% 1 year after HSCT. Of 15 survivors, 73% fully recovered organ function.^16^ Anti‐C5 use requires careful antimicrobial prophylaxis and vaccination planning; indeed, eculizumab therapy showed increased infection rates and infection‐related mortality in children with TA‐TMA.^17^

Another strand of evidence deserving emphasis in TA‐TMA is blockade of the lectin‐pathway. Narsoplimab (a MASP‐2 inhibitor) produced notable response rates in both pediatric and adult patients. In 2022, Khaled et al., studying 28 adults with TA‐TMA treated with narsoplimab, found an improvement in organ function in 74% of patients and an one‐hundred‐day survival after TA‐TMA diagnosis of 68% (94% in responders).^18^ In a large cohort of patients with TA‐TMA high‐risk features, treatment with narsoplimab resulted in a 1‐year overall survival in allo‐HSCT adult and pediatric recipients of 49.5% and 53.2%, respectively, compared with 0%–29% in similar historic cohorts of patients treated only with supportive therapy.^19^ Effectiveness and safety of narsoplimab treatment in patients with TA‐TMA were also observed in real‐world settings.^20^ Narsoplimab was approved by the Food and Drug Administration (FDA) on December 24, 2025, as the first licensed therapy specifically indicated for both adult and pediatric patients with TA‐TMA. All these data support the concept that lectin‐pathway activation is pathophysiologically important in TA‐TMA and that upstream blockade may improve outcomes. Moreover, theoretically, the block of the MBL pathway with narsoplimab leaves the classical pathway intact for the defense against infections.^20^

In those cases of TA‐TMA in which complement activation‐mediated damage appears to play a minor role, the supportive therapies should be the main approach; however, the consideration of complement blocking therapy is not precluded.^15^

## TRIALS AND THE NEAR HORIZON

The field is in an active, rapidly evolving trial phase. Key studies are in progress, such as a randomized multicenter trial of ravulizumab across adult and pediatric cohorts evaluating efficacy, safety, pharmacokinetics, and pharmacodynamics (NCT04543591). A pilot study is ongoing to test pegcetacoplan, a C3 and C3b inhibitor, in 12 patients with TA‐TMA (NCT05148299). In children with TA‐TMA, a multicenter study is testing nomacopan (rVA576), a recombinant small peptide inhibitor of the terminal complement pathway protein C5 (NCT04784455). Successful treatment with iptacopan, an oral inhibitor of complement Factor B, was described in single cases of TA‐TMA.^21^

Studies are underway in adult allo‐HSCT patients to identify plasma biomarkers for early diagnosis of TA‐TMA (NCT06102694) and to evaluate routine screening using harmonized definitions and diagnostic criteria for TA‐TMA during the first 100 days after allo‐HSCT (NCT07059026).

If currently ongoing studies confirm the initial positive signals, we should expect to see evidence‐based guidelines for complement inhibition and early biomarker‐guided therapy in TA‐TMA in the near future.

## CONCLUSION

TA‐TMA exemplifies how better disease definition, mechanistic insight, biomarker science, and targeted therapeutics can converge to change practice rapidly. The past few years have reframed TA‐TMA from an under‐recognized clinical conundrum to a disorder with biological targets and multiple therapeutic candidates. The near future will be defined by trial results that test C5 blockade, lectin‐pathway inhibition, and proximal complement inhibitors, the maturation of biomarker‐driven care, and the tough but necessary comparative studies to determine which patients benefit most from which therapy.

## AUTHOR CONTRIBUTIONS

**Massimo Cugno**: Conceptualization; writing—original draft; writing—review and editing. **Francesco Onida**: Writing—review and editing. **Bernhard Lämmle**: Conceptualization; writing—review and editing.

## CONFLICT OF INTEREST STATEMENT

The authors declare no conflicts of interest.

## ETHICS STATEMENT

Ethical approval was not required for this work as it was conducted on previously published data.

## FUNDING

This work was partially supported by the Piano Nazionale di Ripresa e Resilienza (PNRR), project Malattie Croniche non Trasmissibili (MCnT) ad alto impatto sui sistemi sanitari e socioassistenziali, code PNRR‐MAD‐2022‐12376816, and by the Italian Ministry of Health – Bando Ricerca Corrente, but did not receive any specific grant from funding agencies in the public, commercial, or not‐for‐profit sectors. Open access publishing facilitated by Universita degli Studi di Milano, as part of the Wiley ‐ CRUI‐CARE agreement.

## Data Availability

Data sharing is not applicable to this article as no datasets were generated or analyzed during the current study.
